# Two-dimensional integration approach to teaching cardiovascular physiology: effectiveness and students’ perspectives

**DOI:** 10.1186/s12909-020-02468-9

**Published:** 2021-01-09

**Authors:** Kasiphak Kaikaew, Sarocha Vivatvakin, Maneerat Chayanupatkul, Weerapat Kositanurit, Sekh Thanprasertsuk, Onanong Kulaputana

**Affiliations:** Department of Physiology, Faculty of Medicine, Chulalongkorn University, 1873 Rama IV Road, Pathumwan, Bangkok, 10330 Thailand

**Keywords:** Cardiovascular physiology, Medical students, Integrative teaching, small group discussion, Student performance

## Abstract

**Background:**

Pre-clerkship medical curriculums consist of a series of organ system-based courses and lectures but often lack an integration between organ systems. Such integration could be beneficial for clerkship years and students’ future career. Hence, we aimed to share our process of organising an integrative teaching approach in a large class of pre-clerkship medical students and to reflect the students’ perspective toward the teaching process in this observational study. In addition, we tested effectiveness of this integrative approach compared with the traditional teaching (lecture).

**Methods:**

We organised a two-dimensional (2D)-integrative teaching for 309 students in selected topics of cardiovascular physiology of the medical curriculum of the Faculty of Medicine, Chulalongkorn University, Thailand. The first dimension of integration is the incorporation of physiology of other organ systems into the cardiovascular physiology class. The second is the integration of multiple teaching methods and strategies, including small group discussion, student presentation, wrap-up, quiz, and question-and-answer sessions. Unless opting out, students evaluated this integrative teaching by filling in a questionnaire. The summative scores were also used to determine their comprehensive understandings of the content.

**Results:**

The course evaluation showed that most students (81.9–91.2%) had positive attitudes toward all organised sessions, i.e. this teaching method helps promote their basic and applied physiology knowledge, critical thinking, information searching, presentation, and teamwork skills. In general, students at all performance levels attained higher scores in the summative exam for the 2D-integrative-class–relevant questions (74.4±16.1%) than for the lecture-pertinent questions (65.2±13.6%).

**Conclusions:**

In a large class size of pre-clerkship students, 2D-integrative teaching strategies with careful planning and preparation can be successfully implemented, based on positive attitudes and relatively high summative scores of students in this study. Hence, this comprehensive teaching could be incorporated in current medical curriculums, particularly for the complex learning topics.

**Supplementary Information:**

The online version contains supplementary material available at 10.1186/s12909-020-02468-9.

## Background

As medical knowledge has been continuously expanding, the current trend of the medical curriculum is shifted from the discipline-based learning to the organ system-based learning. The latter learning approach focuses on the knowledge integration of basic medical sciences, such as physiology, anatomy, biochemistry, and other disciplines, to enhance the understanding of the functions and related clinical problems in each organ system [1]. As a result, it potentially wipes out the sense of knowledge fragmentation in medical students [2, 3]. Additionally, several studies reported that students who received the integrated teaching had a better academic performance than the traditional teaching method [2, 4, 5]. Despite the abundance of interdisciplinary integration of knowledge in one organ system, the incorporation of knowledge from different organ systems may remain insufficient in the organ system-based teaching method. We realised the potential lack of knowledge integration in this dimension and thus implemented it in our course.

Most Thai medical students these days are the members of the Generation Z (Gen Z), i.e. born between 1995 and 2012 [6, 7]. Gen Z students do not typically tolerate spending a long time listening to lectures of basic knowledge. What they are eager to know is where the particular pieces of knowledge they are learning can be placed in and beneficial to their future career [6, 8]. Thus, the integration of clinical scenarios into basic medical science teaching was carried out in our Cardiovascular System I course. In addition, multiple teaching methods, including self-directed learning before class, small group discussion, student presentation, wrap-up lecture, quiz, and question and answer (Q&A) sessions, were employed to promote the goal-directed learning nature of our Gen Z medical students.

Herein, we report a two-dimensional (2D)-integrative teaching in the topic of cardiovascular physiology at the Faculty of Medicine, Chulalongkorn University, Bangkok, Thailand. This topic is in the Cardiovascular System I course during the second year of the six-year Doctor of Medicine curriculum. The first dimension of the integration is the incorporation of physiology of other organ systems into the cardiovascular physiology class or the physiology knowledge integration. The second is the application of multiple teaching methods and strategies in this single learning topic containing the correlation between cardiovascular physiology and clinical experience or the teaching method integration. The multi-faceted learning outcomes including the basic knowledge in cardiovascular physiology and the relevant clinical contexts, the critical thinking skill, the professional development aspects (e.g. learning how to learn, information searching skill, presentation skill, etc.), as well as the leadership and teamwork skills were expected in the students under this teaching approach. The main objectives of this article were to share our experience in the administrative process and implementation of integrative teaching and to reflect the students’ perspectives toward this teaching approach based on the course evaluation. In addition, the effectiveness of this integrative teaching approach, compared with the traditional teaching (lecture), was also evaluated using students’ summative scores.

## Methods

### Study population and study design

**Fig. 1:**
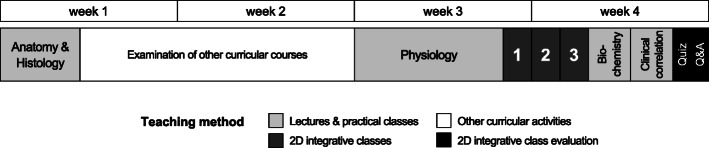
Cardiovascular System I course schedule and teaching methods. Upper boxes demonstrate the course schedule that 1 week contains 5 study days. Lower boxes demonstrate general themes and topics of which filled colour indicates teaching methods. Numbers 1, 2, and 3 indicate each 2D-integrative session

The current medical education at Chulalongkorn University is a six-year undergraduate curriculum consisting of 3 pre-clerkship and 3 clerkship years. Pre-clerkship education mainly focuses on basic medical sciences, such as anatomy, physiology, pharmacology, and pathology. Curriculum in the second year covers the principles of anatomy, biochemistry, and physiology, which are divided into multiple organ system-based courses including neuroscience, musculoskeletal, respiratory, cardiovascular, gastrointestinal, urinary, endocrine, and reproductive systems. The Cardiovascular System I was taught in the second pre-clerkship year at the Faculty of Medicine, Chulalongkorn University, Bangkok, Thailand during August 8th–9th and August 22nd – September 4th, 2019 (Fig. 1). All learning topics, teaching methods, duration of each class, and responsible departments are listed in Additional file 1. In brief, all basic principles of the cardiovascular system were taught by the traditional teaching method (lecture) in a total of 24.5 h. The lectures were hosted in a 320-seated lecture theatre and were recorded and posted in the online database of the Faculty for further self-study by students when needed.

The study population of this analytic observational study was 309 second-year medical students in the academic year of 2019. Since the intervention of this study (the 2D-integrative classes) was part of the course, we enrolled all students in the study. However, if the students opted out, their data were excluded from statistical analyses.

### Intervention (2D-integrative classes)

**Fig. 2:**
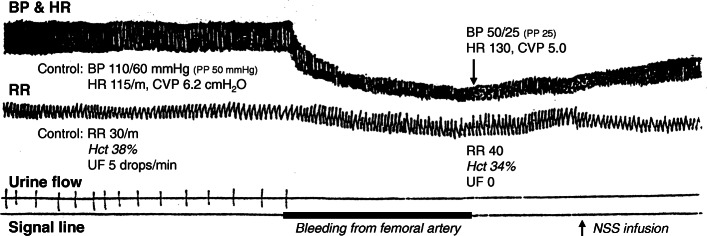
A representative image of monitored tracings in the condition of arterial haemorrhage. This image demonstrates 4 tracings from top to bottom as follows. **1)** Arterial pressure tracing, monitored at the right carotid artery, indicates systolic BP (upper borders of signal lines), diastolic BP (lower borders of signal lines), and HR (frequency of signal lines). **2)** Respiratory tracing, recorded from chest wall movement, indicates tidal volume and RR. **3)** Urine flow tracing, monitored at the right ureter, indicates urine output (drop per each indicated line). **4)** The signal line indicates an intervention performed to an animal at an indicated time, i.e. arterial bleeding from the right femoral artery. Of note, CVP was determined at the left external jugular vein and values at an indicated time were shown in the figure. Abbreviations: BP (blood pressure), CVP (central venous pressure), Hct (haematocrit), HR (heart rate), NSS (normal saline solution), PP (pulse pressure), RR (respiratory rate), and UF (urine flow)

At the end of the academic year 2018, we (the faculty members of the Department of Physiology, Faculty of Medicine, Chulalongkorn University) reviewed the students’ feedback and comments on teaching and learning in the Cardiovascular System I course, and hence we planned to organise the integrative teaching approach for the topic of cardiovascular physiology disturbances in the academic year of 2019. These integrative classes were structured according to students’ feedback and inputs from academic staffs in the Department. The integrative classes focused on the small group discussion on the topic of cardiovascular physiology disturbances in which a problem-focused learning approach was implemented [9]. The contents of the discussion included animal experiments of various cardiovascular conditions. We used dogs in these models. The experimental procedures were video-recorded several years ago for future classes in the attempt to minimise animal use. In each experimental condition, several questions were posted for students to discuss with their peers in a small group. All questions were generated and updated annually by several instructors from the Department with expertise in different organ systems in order to integrate knowledge from multiple systems. Examples of experimental conditions with multiple sub-experiments and questions for the academic year 2019 are demonstrated in Table 1. For instance, upon an alteration by arterial haemorrhage (experiment 2.1 in Table 1), various cardiovascular and systemic responses were monitored (Fig. 2). Points for discussion in this haemorrhagic shock condition include an integration of multiple organ systems, such as respiratory system: adaptation of lung mechanics; urinary system: alteration of renal blood flow, glomerular filtration, and tubular reabsorption; endocrine system: the effect of renin-angiotensin-aldosterone system; neuroscience: autonomic responses upon the sudden drop of systemic mean arterial pressure.

**Table 1:** Examples of experimental conditions and questions for small group discussion

**Experiment 1. Effects of the venous blood loss**	
*1.1) Early effects of the venous blood loss* 1.1.1) After draining blood from the venous system (femoral vein), what are the most probable changes in the parameters including BP, PP, HR, CVP, RR, Hct, and UF? Explain the mechanisms of these changes. 1.1.2) A 20-year-old man presented with severe haemorrhage. His BP was 95/65 mmHg and his HR was regular at 160 BPM. A physician chose to treat him with a negative inotropic drug in an attempt to lower his HR to the normal baseline. Is his management suitable? Explain with reasons. *1.2) Late effects of the venous blood loss (15 min later)* Fifteen minutes after the onset of bleeding, BP began to rise, and HR began to decrease but still did not reach the baseline. Hct declined and urine flow increased, but again did not reach the baseline. What are the probable mechanisms that could explain these changes?	
**Experiment 2. Effects of arterial blood loss and the treatments by normal saline infusion and blood transfusion**	
*2.1) Effects of arterial blood loss* What are the differences in terms of cardiovascular responses between arterial bleeding from femoral artery (this experiment) and venous bleeding from femoral vein (the prior experiment)? Explain the mechanisms of these differences. *2.2) Effects of normal saline infusion after arterial bleeding* What are the most probable changes in the parameters including BP, HR, CVP, Hct, and UF after the administration of normal saline solution of the same quantity with the amount of blood loss? *2.3) Effects of blood transfusion as a compensation for arterial bleeding* (This experiment was performed 10 min after normal saline infusion. The blood for transfusion was obtained from the femoral vein in experiment 1 and was transfused via the femoral vein.) 2.3.1) Ten minutes after normal saline infusion, BP decreased and HR increased. Explain the probable mechanisms of these changes. 2.3.2) What are the probable changes in terms of cardiovascular responses after blood transfusion compared with normal saline infusion?	
**Experiment 3. Effects of nitroglycerin administration**	
3.1) After an administration of nitroglycerin, what are the most probable changes in the parameters including BP, PP, HR, CVP, RR, and UF? 3.2) What should be the mechanisms of action of nitroglycerin on the cardiovascular system that can best explain all the parameter changes mentioned above?	
**Experiment 4. Effects of adrenaline administration**	
4.1) What are the most probable effects of adrenaline administration on the parameters including BP, HR, and RR at an indicated time according to the recorded tracing? Explain the mechanisms of these changes. 4.2) State the effects of adrenaline on UF and explain the mechanisms of this change.	
**Experiment 5. Effects of different doses of dopamine administration**	
5.1) State the effects of a gradual increase in dopamine intravenous infusion from 1 μg/kg/min to 20 μg/kg/min on BP, HR, RR, and UF. Compare the effects that occurred while receiving dopamine in different doses. 5.2) Explain the mechanisms of action of dopamine that can best explain the results of this experiment.	
**Experiment 6. Vagus nerve stimulation before and after atropine administration**	
6.1) Explain the effects of right vagus nerve stimulation on BP, HR, and RR before an intravenous administration of atropine. 6.2) During the intravenous atropine administration, what are the expected changes in BP and HR? Explain the probable mechanisms. 6.3) Explain the effects of right vagus nerve stimulation on BP, HR, and RR after an intravenous administration of atropine.	
**Experiment 7. Effects of nitroglycerin after atropine administration**	
What are cardiovascular responses to the intravenous injection of nitroglycerin after atropine administration? Are they similar to the responses before atropine administration (experiment 3)? Explain the probable mechanisms.	
**Experiment 8. Effects of adrenaline after atropine administration**	
What are cardiovascular responses to the intravenous injection of adrenaline after atropine administration? Are they similar to the responses before atropine administration (experiment 4)? Explain the probable mechanisms.	

Abbreviations: *BP* blood pressure, *BPM* beats per minutes, *CVP* central venous pressure, *Hct* haematocrit, *HR* heart rate, *PP* pulse pressure, *RR* respiratory rate, *UF* urine flow

Before the start of the Cardiovascular System I course, teaching materials and guided answers of all experimental conditions and questions were distributed to a total of 22 facilitators (17 academic staffs and 5 medical doctors who work as teaching assistants in the Department). Furthermore, the cardiovascular experts organised a 4-h session of lecture and seminar for the new facilitators and the facilitators who needed knowledge refreshment. This session was organised to ensure that all facilitators were confident to guide and facilitate the small group discussions.

Concerning the course management for students, all experimental conditions and questions were given to the students on the first day of the course. This provided them with opportunities to read the questions before the discussion class. To ensure that the students had already learned all the essential knowledge, the discussion classes were scheduled during the third and fourth weeks of the course (Fig. 1). Students watched the pre-recorded videos of the experimental procedures for approximately 30 min before they were divided into small groups. A total of 309 students were divided into 4 major groups, which they were then subdivided into 3 smaller groups of 25–26 students as it was more practical for facilitators to observe the student learning behaviours. Round table discussion of 5–7 students was encouraged since it provided better opportunities for each student to participate. One to two facilitator(s) were assigned to each small group (25–26 students) to guide the discussion process and to clarify any unclear points of the contents. The total time for discussion was 2 h. Students were instructed to discuss and answer all questions within the discussion period and the facilitators must ensure that all students under their responsibility understood all key concepts. Although every student had to answer all posted questions, each group of students was assigned a number of questions to present their answers and physiological explanation to the major group (77–78 students with 3–5 facilitators) after the discussion period. The presentation part took approximately 1 h. As a positive reinforcement measure, each student was observed by the assigned facilitators who gave points for their attendance, participation, and discussion and presentation skills.

After the presentations were concluded in all 4 major groups, students returned to the main classroom for the wrap-up session. The wrap-up session was organised in the form of a short lecture that summarised the essential contents of the experiments by a cardiovascular expert from the Department in a 320-seated lecture theatre. The purpose of this session was to ensure that all students understood the core contents in each discussion session. The students could interact with the content expert directly in this wrap-up session and were encouraged to send any further questions to the instructor after the session through available online platforms, i.e. by e-mailing, private messaging in the Facebook Messenger, or posting in the closed Facebook group containing all classmates and academic staffs of the Department.

After the last discussion session, there was a cardiovascular physiology quiz for the students to test their knowledge and motivate their further self-study. Since the course coordinating committee considered the quiz as an essential part to timely assess students’ knowledge and provide feedbacks, we strongly encouraged the students to participate in this quiz session by arranging the quiz settings to mimic those in the summative exam, and thus 308 students participated. The quiz contained 30 multiple-choice questions (MCQ with 4 choices and single best answers). Twenty-four questions were based on the learning contents of the discussion classes (Additional file 2), while the remaining 6 questions were based on other related lectures of the course. Students had 30 min to complete the quiz. Then, there was a Q&A session just after the quiz. This session was prepared by multiple physiology experts, consisted of 2 parts, and lasted for one and a half hours. The first part was to provide the correct answers to the quiz, along with the explanation for the answers. In the second part, students could dispute if they disagreed with the answers or could ask any further questions regarding course contents. The students’ participation in the Q&A session was voluntary and their attendance was not recorded.

### Course evaluation

At the end of the course, the students were asked to anonymously fill a course evaluation questionnaire regarding their attitudes toward the small group discussion, the wrap-up, the quiz, and the Q&A sessions. The evaluation form was divided into 3 parts. The first part was the five-point Likert scale survey which was consisted of 10 statements (Table 2). The Likert scale consisted of a scale of 1 to 5: 1, strongly disagree; 2, disagree; 3, neither agree nor disagree; 4, agree; and 5, strongly agree. The higher the score implied a more positive attitude toward that statement. The second part contained questions regarding the quiz and the Q&A sessions. Students were asked whether they agreed that these sessions improved their understanding of the learning content and whether the quiz session should be carried on in the next academic year. The last part of the questionnaire was an open-ended question for students’ comments and suggestions. Students’ feedback was summarised independently by two assessors. The course evaluation was entirely voluntary and students could opt in or opt out to answer any part of the questionnaire.

**Table 2:** Course evaluation results regarding the small group discussion sessions

Evaluation statements	Agree	Uncertain	Disagree
1) Overall satisfaction with the discussion	219 (88.7%)	27 (10.9%)	1 (0.4%)
2) Small group discussion improves your understanding of lecture contents.	215 (86.3%)	30 (12.1%)	4 (1.6%)
3) Small group discussion helps improving your critical thinking and reasoning skills.	220 (88.7%)	26 (10.5%)	2 (0.8%)
4) Participating in the small group discussion classes provides an opportunity for you to practice teamwork and presentation skills.	204 (81.9%)	43 (17.3%)	2 (0.8%)
5) Small group discussion encourages you to seek further and deeper knowledge.	207 (83.1%)	39 (15.7%)	3 (1.2%)
6) You search for additional information beyond what was learned in the lecture classes for the small group discussion.	206 (82.7%)	40 (16.1%)	3 (1.2%)
7) The number of facilitators per group is appropriate.	203 (81.5%)	31 (12.5%)	15 (6.0%)
8) The wrap-up session after the discussion class is essential.	227 (91.2%)	20 (8.0%)	2 (0.8%)
9) In the following year, you still want to have the wrap-up session after the discussion.	220 (88.4%)	26 (10.4%)	3 (1.2%)
10) You have gained teamwork skills during the discussion class.	218 (87.9%)	28 (11.3%)	2 (0.8%)

Data are presented as the number of responses and the percentage. “Agree” includes the responses strongly agree and agree whereas “Disagree” include the responses strongly disagree and disagree on the five-point Likert scale

The perspectives of the academic staffs, the students’ comments and summative scores (detail in the section below) were discussed in the meetings of the Department staffs and in the Cardiovascular System course coordinating committee. After the brainstorming sessions, suggestions and comments of all academic staffs and committees were recorded in the meeting minutes which will be used for improving the teaching approach and materials in the next academic year.

### Knowledge assessment and item analysis

The knowledge assessment (summative exam) of the Cardiovascular System I course took place on October 15th, 2019 (~ 6 weeks after the end of the course), which was a routine summative exam in our medical curriculum. The exam consisted of 110 questions (MCQ with 5 choices and single best answers), of which 63 questions were related to cardiovascular physiology taught by the traditional learning method (lecture) or by the 2D-integrative classes. Of note, no question was exactly the same as the quiz held at the last session of the course. The remaining 47 questions were related to cardiovascular anatomy or biochemistry and were excluded from the analysis since those questions were not relevant to our research focus. For these 63 questions, three instructors independently categorised whether the questions were related and applicable to the physiological knowledge taught in the 2D-integrative sessions. As a result of an agreement of at least 2 instructors, 16 questions were categorised into 2D-integrative class–relevant questions and 47 questions were categorised into lecture-pertinent questions.

The 63 MCQ items were analysed for acceptability, difficulty, and discrimination indices. Acceptability index (AI, so-called the test-centred item judgement) was assessed by the Ebel method [10, 11]. In brief, three instructors independently determined the level of difficulty (easy, appropriate, or difficult) and relevance (essential, important, acceptable, or questionable) of each item in a random order. The expected percentage of correct answers was classified into the 3× 4 difficulty-by-relevance matrix table. The AI of each item was calculated by an average of the expected percentages from three instructors. The median of the expected percentage of correct answers was used to classify each item into the high or low AI items. Difficulty index (DIFF) was calculated as a proportion of the students who chose the correct answer to the total number of responders, ranging from 0 to 1. The optimal DIFF ranges between 0.2 and 0.8, DIFF < 0.2 was considered a difficult item, and DIFF > 0.8 was considered an easy item [12]. Discrimination index (DI) was calculated by a difference in the proportion of the top 27% and the bottom 27% of performers of the total physiology items who answered that question correctly. DI ranges from − 1 (only all the bottom 27% responded to an item correctly) to 1 (only all the top 27% responded to an item correctly), of which DI ≥ 0.40 was considered a very good item, 0.20–0.39 was considered a reasonable item, and < 0.20 was considered a poor item [13]. Of the 63 questions, there were two items with a negative DI implying their poor quality and thus were excluded from the statistical analyses. Of note, these two questions were considered difficult (low-AI) lecture-pertinent items.

To identify if the 2D-integrative session was effective for the students at all performances, we categorised the students’ performances by their overall scores of 61 questions into three subgroups by percentile ranks: the high-performance group (percentile rank 0.67–1.00), the average-performance group (percentile rank 0.34–0.66), and the low-performance group (percentile rank 0.00–0.33). Of note, one student missed the summative exam due to personal reasons and one student opted out of the study. Hence, 307 students were enrolled in this part of the statistical analysis.

### Statistical analysis

Result calculation and statistical analysis were performed using Microsoft Excel (version 16.0.12827.20236) and GraphPad Prism (version 8.4.3). Unless indicated, data are shown as mean±SD. The differential distributions of DIFF and DI were determined by Chi-square test. The difference in overall scores between lecture-pertinent items and integrative-class items were determined by paired *t* test. The difference in physiological scores among students’ performance subgroup, AI classification, and question category was analysed by three-way ANOVA with repeated measures, followed by appropriate *post-hoc* tests: two-way ANOVA and Tukey test.

## Results

**Fig. 3:**
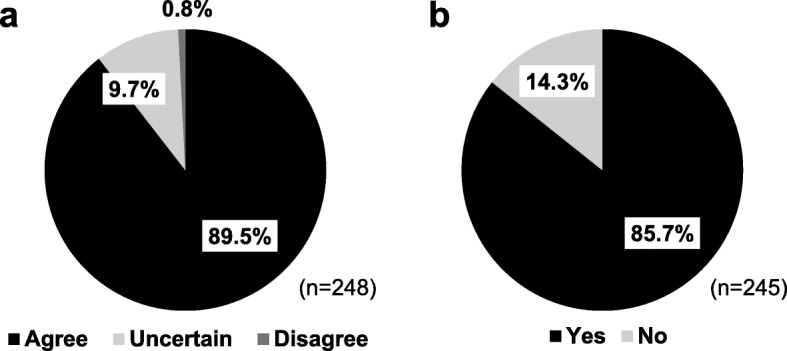
Students’ responses regarding the quiz and Q&A sessions. At course evaluation, students were asked to rate whether **a** the quiz and Q&A sessions make them understand the cardiovascular physiology better and **b** the quiz session should be carried on the next academic year. Data are presented as the percentage of responses to the total number of responders to the question

Of 309 students enrolled in the Cardiovascular System I course, 248 students (80.3%) participated in the course evaluation whereas 61 students (19.7%) did not return the evaluation form. In general, more than 80% of students agreed with all 10 statements of satisfaction regarding the small group discussion and the wrap-up sessions (Table 2). In addition, 222 students (89.5%) believed that the quiz and Q&A sessions helped them understand the content better (Fig. 3a) and 210 students (85.7%) were in favour of continuing the cardiovascular physiology quiz in the next academic year (Fig. 3b). Students’ open-ended feedbacks were categorised into positive and negative comments for the ease of outcome interpretation (Table 3). The positive comments were those comments related to the benefits of the discussion, whereas the negative ones included the drawbacks of the discussion or points for improvement.

**Table 3:** The positive and negative open-ended feedbacks of the discussion session

	Positive comments	Negative comments
**Group members**	• Improving their comprehension due to interactive discussion and opinion sharing among group members (30 responses) • Promoting students to express their ideas which raises their self-confidence (5 responses) • Immediate feedback among peers during discussion (3 responses)	• Too fast discussion to catch up with for some students (3 responses) • Too large number of students in each group (2 responses) • Only some students dominating the discussion (1 response)
**Facilitators**	• Immediate responses to students’ questions (10 responses) • Clearly explaining student’s questions and pointing out important contents (5 responses) • Encouraging student to ask questions (5 responses)	• Too few facilitators in each group (8 responses) • Variation of contents perceived by students due to different facilitators (8 responses)
**Contents**	• Integrating and reviewing all knowledge from lectures and applying theory to explain clinical scenarios (23 responses) • Facilitating critical thinking skills (11 responses) • Motivating students’ self-learning process (5 responses) • Highlighting key points to help students memorising and understanding contents (4 responses)	• Difficulty in understanding of some contents (6 responses) • Uncertainty in the accuracy of contents discussed and presented by other students (2 responses)
**Presentation**	• Additional explanation by facilitators during the presentation (2 responses)	• Students’ stress during the presentation and ignorance of other contents that they are not assigned to present (7 responses)
**Wrap-up**	• Summarising the essential knowledge (3 responses)	• Too short time of wrap-up session (2 responses)

**Fig. 4:**
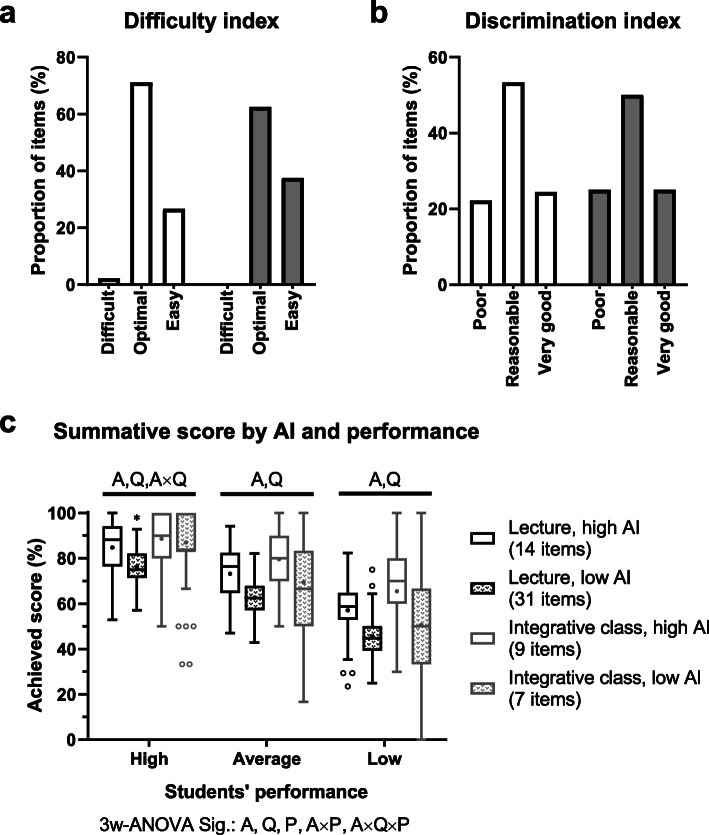
Item analysis and summative scores. Cardiovascular physiology knowledge was assessed by the summative exam of 61 multiple-choice questions with single best answers, consisting of 45 lecture-pertinent questions and 16 items of the 2D-integrative-class–relevant questions. **a** Difficulty index and **b** discrimination index of the summative exam are presented as a proportion of the questions to the total number in that category. **c** Students’ summative scores are categorised by student performance tertiles of the overall physiological exam score, test-centred acceptability index (AI), and question category (lecture-pertinent or 2D-integrative-class–relevant learning method). Box-and-whisker plot presents the Tukey method of which boxes indicate interquartile range (IQR), whiskers indicate 1.5 times IQR, lines in the box indicate the median, and plus signs in the box indicate the mean. Blank dots indicate individual scores higher than the upper whisker or lower than the lower whisker. Letters and symbols indicate statistical significance: A (acceptability index), Q (question category), P (students’ performance tertile), A×P (interaction of A and P), A×Q (interaction of A and Q), A×Q×P (interaction of A, Q, and P), and * (difference from the other subgroups by *post-hoc* Tukey test)

Test-centred AI and learning method relevance were applied to classify the 61 MCQ items into 4 categories: 14 high-AI lecture-pertinent items, 31 low-AI lecture-pertinent items, 9 high-AI 2D-integrative-class–relevant items, and 7 low-AI 2D-integrative-class–relevant items. Students’ knowledge of the cardiovascular physiology were assessed by the summative exam of which item analysis showed no significant difference in DIFF (*P* = 0.62) and DI (*P* = 0.97) between lecture-pertinent items and 2D-integrative-class–relevant items (Fig. 4a–b). In general, students achieved higher scores in the 2D-integrative-class–relevant items (74.4±16.1%) than in the lecture-pertinent items (65.2±13.6%, *P* < 0.001). Intriguingly, students at all performance levels attained higher scores in the questions related to the 2D-integrative classes than in the lecture-pertinent questions and higher scores in high-AI items than in low-AI items (Fig. 4c).

## Discussion

We have shown how we successfully organised the 2D-integrative class for the topic cardiovascular physiology in a pre-clerkship undergraduate medical curriculum. Most students reflected that our 2D-integrative teaching method improved their understandings of the learning contents, increased their critical thinking skills, motivated self-learning processes, built teamwork and presentation skills, and helped them evaluate their understandings. In addition, the result showed that students at all performance levels had higher scores in the 2D-integrative-class–relevant questions than in the lecture-pertinent questions, when the acceptability index of the questions was controlled. This could be extrapolated that teaching by the 2D-integrative method, including problem-focused learning approach, could result in better academic performance than the traditional teaching (lecture) method, which was in agreement with previous studies [2, 14, 15]. Since we previously demonstrated that positive attitudes are keys to the study performance for Thai medical students [16], the positive reflections toward the 2D-integrative teaching might account for the higher academic performance.

Based on the course evaluation results, most students had positive attitudes toward the small group discussion sessions. Although not the entire class participated in the course evaluation, we assume that 80.7% of students who voluntarily filled in the questionnaire were reasonably representative of the class. The reason students had positive attitudes toward the discussion sessions was probably the integrative nature of the content. They had to apply basic physiology knowledge in many organ systems in order to answer the questions with a reasonable explanation. Thus, with less than 2 years of experience in the field of medical physiology, working in a group might facilitate their problem-solving process of such integrative problems. Group discussion is a teaching method that was introduced for a long time in order to influence the students to be more proactive, more interactive, and more enjoyable [17–21]. This teaching style was also reported to be associated with greater integration of new information with pre-existing knowledge [19]. Therefore, small group discussions should be implemented especially when the learning content is integrative.

The wrap-up lecture session was also believed, by most students, to play a pivotal role in the overall discussion session and should also be included in the next-year course. As the discussion content was quite integrative in terms of physiology knowledge of multiple organ systems, we believed that the summary of the key concepts was important as this would at least in part reduce the students’ frustration caused by their perception of information overloading. This key concept summary was thus shared with the students in our wrap-up session. Furthermore, another purpose of the wrap-up lecture was to ensure that core contents were not missed due to the variation of the facilitators. One descriptive study found that the wrap-up session after a problem-based learning approach helped improve students’ understanding of the learning content and recommended the wrap-up session to be implemented in the medical education curriculum [22]. However, the issue regarding the benefits of the wrap-up session should be systematically studied in the future.

Also, most students had positive responses to the quiz and Q&A sessions. The concept of our quiz resembled the general concept of formative assessment, which helped students evaluate their own academic performance, i.e. what they had already known and what they needed to know more, and consequently led to a significant benefit to student learning [23–27]. The Q&A session probably improved their understanding by addressing the problems that came up in prior sessions, including the traditional lectures, small group discussion, wrap-up sessions, and the quiz. In other words, the learning experience in these last sessions was constructed mainly by the integration of contents from all teaching methods. In our viewpoint, the Q&A session is quite resembling the wrap-up lecture session in terms of key concept summary, but with more orientation to the students’ problems, more direction to their goal, more teacher-student interaction, and more integration of teaching method experience.

Regarding students’ satisfaction with the number of facilitators per group, a majority of students (82%) were satisfied with one to two facilitators, whereas 6% were not. However, these 6% are relatively large, comparing to the evaluation results in other aspects. We had realised this problem from the classes in previous years and tried to handle it by assembling three small groups into one major group; hence, the facilitators from three small groups could work together. The evaluation results in the present academic year, however, still reflected that the number of the facilitators might not be enough to timely address all students’ questions during the group discussion. Increasing the number of facilitators per group in the subsequent academic year is under consideration, although achieving this goal is a challenge.

Additionally, how long the duration of the discussion session should last is worth debating. Some students believed the discussion took too long, while others believed they did not have enough time during the discussion to understand all contents. This discrepancy was presumably related to the variation of students’ baseline knowledge, which appeared to be a main limitation in our teaching method. Mixing the well-performed with the poorly performed students in the same group should help improve knowledge of the poorly performed ones and improve the tutoring abilities of the well-performed ones. On the other hand, the well-performed students might lose their opportunity to understand some advanced content. The poorly performed students might also lose their attention while listening to the explanation from the well-performed ones. Based on our viewpoint, the topic about the discrepancy of students’ baseline knowledge in the discussion group is thus controversial and the systematic study should be conducted to circumvent this controversy.

## Conclusions

We successfully organised the 2D-integrative teaching in cardiovascular physiology, including the integration of physiology knowledge of multiple organ systems and the integration of multiple problem-focused learning methods, including small group discussion, wrap-up lecture, quiz, and Q&A sessions. Most students reflected that this learning experience helps promote their basic and applied physiology knowledge, critical thinking, information searching, presentation, and teamwork skills. Despite a greater invested manpower, this 2D-integrative teaching approach should thus be applied in the current medical education curriculum whenever suitable, particularly for the complex topics that require extent and depth of understanding.

## Supplementary Information

**Additional file 1.** The table indicating topic outline of the Cardiovascular System I course.

**Additional file 2.** The quiz containing 24 multiple-choice questions with single best answers related to the 2D-integrative classes.

## Data Availability

The datasets generated and/or analysed during the current study are not publicly available but are available from the corresponding author on reasonable request.

## Abbreviations

AI: Acceptability index
DIFF: Difficulty index
DI: Discrimination index
Gen Z: Generation Z
MCQ: Multiple-choice questions
Q&A: Question and answer
2D: Two-dimensional
