# Supplementary material for: Two-dimensional integration approach to teaching cardiovascular physiology: effectiveness and students’ perspectives
Source: BMC Med Educ. 2021 Jan 9;21:43. doi: 10.1186/s12909-020-02468-9 (PMC7796487; doi:10.1186/s12909-020-02468-9)
Supplement: Supplementary file 1 — Additional file 1. The table indicating topic outline of the Cardiovascular System I course. [file 12909_2020_2468_MOESM1_ESM.pdf]

Learning Topic Outline of the Course
Cardiovascular System I, Academic Year 2019
Faculty of Medicine, Chulalongkorn University

Topics	Teaching methods	Hours	Department of instructor(s)
Anatomy of heart & great vessels	Lecture	1	Anatomy
Anatomy of heart & great vessels	Practicum	3	Anatomy
Thorax topography	Lecture	1	Anatomy
Thorax topography	Practicum	2	Anatomy
Histology of heart & vessels	Lecture	1	Anatomy
Histology of heart & vessels	Practicum	3	Anatomy
Review of Anatomy & histology	Conference	3	Anatomy
Physical & electrical properties of the heart	Lecture	2	Physiology
Cardiac cycle & regulation of the heart	Lecture	3	Physiology
Heart contraction, extrasystole, vagus nerve & heart block	Conference	3	Physiology
Basic EKG	Lecture	1	Physiology
Basic EKG	Practicum	3	Physiology
Circulatory dynamics	Lecture	2	Physiology
Circulatory control	Lecture	2.5	Physiology
Microcirculation	Lecture	1	Physiology
Microcirculation	Practicum	3	Physiology
Regional Circulation	Lecture	1.5	Physiology
Pathophysiology of shock & heart failure	Lecture	2.5	Physiology
Blood pressure measurement	Practicum	3	Physiology
Basic physical examination of the heart	Practicum	2	Physiology
Exercise Physiology	Lecture	1	Physiology
Physical activity & fitness	Lecture	1	Physiology
Physical activity & fitness	Practicum	4	Physiology
Disturbances of CVS 1	Integration	3	Physiology
Disturbances of CVS 2	Integration	3	Physiology
Disturbances of CVS 3	Integration	3	Physiology
Biochemistry of the heart	Lecture	1	Biochemistry
Biochemistry of the blood	Lecture	1.5	Biochemistry
Hemostasis	Lecture	1.5	Laboratory Medicine
Hemostasis	Practicum	1.5	Laboratory Medicine
Conference on cardiovascular cases	Conference	3	Physiology
CVS quiz	Quiz	0.5	Physiology
Question & answer	Q&A	1	Physiology

**Summary**	Lecture	24.5	hours
	Non-lecture	44	hours
	Self-directed learning	48	hours (estimated, not in the above table)
