# Supplementary material for: Two-dimensional integration approach to teaching cardiovascular physiology: effectiveness and students’ perspectives
Source: BMC Med Educ. 2021 Jan 9;21:43. doi: 10.1186/s12909-020-02468-9 (PMC7796487; doi:10.1186/s12909-020-02468-9)
Supplement: Supplementary file 2 — Additional file 2. The quiz containing 24 multiple-choice questions with single best answers related to the 2D-integrative classes. [file 12909_2020_2468_MOESM2_ESM.pdf]

The CVS quiz showing items related to the 2D-integrative classes

Question: Which of the following changes is observed with downward head tilt of the dog?
A. Decreased venous return
B. Decreased myocardial contraction rate
C. Decreased stimulation of the cardio-inhibitory center
D. Decreased stimulation of stretch receptors at carotid sinuses and aortic arch
Answer: B

Question: A 1-year-old boy presents with cough, dyspnea, and fever. He is diagnosed with laryngotracheobronchitis and has suffered from the obstruction in the upper airway for a short period of time. During the event, 1) which of the following is the expected result of arterial blood gas analysis and 2) which receptors are stimulated?
A. 1) Normal pH and decreased PO_2, 2) Baroreceptors
B. 1) Decreased pH and normal PO_2, 2) Baroreceptors
C. 1) Increased pH and normal PO_2, 2) Chemoreceptors
D. 1) Normal pH and decreased PO_2, 2) Chemoreceptors
Answer: D

Question: Which of the following is the cardiovascular response after baroreceptors are stimulated?
A. Increased heart rate
B. Decreased afterload
C. Decreased vagal tone
D. Increased myocardial contractility
Answer: B

Question: When the splanchnic nerve that supplies adrenal medulla is stimulated, which of the following is an expected change?
A. Decreased heart rate
B. Decreased urine flow
C. Decreased myocardial contractility
D. Decreased left-ventricular ejection fraction
Answer: B

Question: A 20-year-old man presents with palpitation (160 bpm of pulse rate). Supraventricular tachycardia is diagnosed. A physician performs carotid sinus massage. What is the physiologic mechanism of this procedure to terminate the tachycardia?
A. Increased vagal tone
B. Stimulation of the vasomotor center
C. Inhibition of the cardio-inhibitory center
D. Decreased stimulation of carotid baroreceptors
Answer: A

Question: A 20-year-old man with skin abscess at the right forearm requires an incision and drainage. If a surgeon had cut his skin without administering local anesthesia, which of the following responses would have occurred?

A. Decreased heart rate
B. Increased vagal tone
C. Increased sympathetic tone
D. Decreased myocardial contractility

Answer: C

Question: In an experimental dog with double vagotomy, what should be the most probable response of heart rate as a result of carotid sinus stimulation?

A. Increased
B. Decreased
C. Unchanged
D. Unpredictable

Answer: C

Question: Which of the following is the most appropriate medication to increase heart rate in a heart transplant patient?

A. Sympatholytic agent
B. Sympathomimetic agent
C. Parasympatholytic agent
D. Parasympathomimetic agent

Answer: B

Question: Which of the following is the primary mechanism of hypotension in a patient with a large amount of pericardial effusion?

A. Decreased venous return
B. Increased left ventricular afterload
C. Decreased central venous pressure
D. Impaired left ventricular systolic function

Answer: A

Question: Which of the following changes of cardiovascular parameters most likely occur in response to an acute occlusion of the inferior vena cava? (VR = venous return, SV = stroke volume, CO = cardiac output, BP = blood pressure, and HR = heart rate)

A. VR ↓, SV ↓, CO ↓, BP ↓, HR ↑
B. VR ↑, SV ↑, CO ↑, BP ↑, HR ↓
C. VR ↓, SV ↑, CO ↓, BP ↓, HR ↑
D. VR ↑, SV ↓, CO ↑, BP ↑, HR ↓

Answer: A

Question: Which symptom or sign should **NOT** be a result of severe aortic stenosis?

A. Syncope
B. Chest pain
C. Heart failure
D. High pulse pressure

Answer: D

Question: During the stimulation of the aortic nerve with low electrical current, 1) which receptors are stimulated and 2) what is the expected change in heart rate (HR)?

A. 1) Aortic arch baroreceptors, 2) Decreased HR
B. 1) Aortic body baroreceptors, 2) Increased HR
C. 1) Aortic arch chemoreceptors, 2) Decreased HR
D. 1) Aortic body chemoreceptors, 2) Increased HR

Answer: A

Question: Which of the following stimulations and effects result in an increase of PR interval on an electrocardiogram?

A. Stimulation of left vagus nerve due to its predominant effect at the SA node
B. Stimulation of left vagus nerve due to its predominant effect at the AV node
C. Stimulation of right vagus nerve due to its predominant effect at the SA node
D. Stimulation of right vagus nerve due to its predominant effect at the AV node

Answer: B

Question: Which of the following is **LEAST** likely the early finding in a dog after a destruction of aortic valve?

A. Increased pulse pressure
B. Increased systolic pressure
C. Increased diastolic pressure
D. Increased preload of the left ventricle

Answer: C

Question: Which of the following statements is **FALSE** regarding atrial fibrillation?

A. Atrial systole phase is present.
B. Pulse rate is irregularly irregular.
C. It causes inconsistent pulse intensity.
D. Blood pressure varies from beat to beat.

Answer: A

Question: Which of the following statements is true regarding ventricular fibrillation?

A. It has irregular pulse rate.
B. Blood pressure is not measurable.
C. Ventricular fibrillation is less severe than atrial fibrillation.
D. After electrical stimulation of ventricles is stopped, sinus rhythm resumes.

Answer: B

Question: Which of the following should initially be given to a patient with hypovolemic shock to maintain microcirculation perfusion?

A. Colloid solution
B. Blood transfusion
C. Isotonic saline solution
D. Either isotonic solution or blood transfusion

Answer: C

Question: Which of the following is **NOT** a compensatory mechanism in response to a blood loss?

A. Increased sympathetic outflow
B. Increased glomerular filtration rate
C. Decreased baroreceptor stimulation
D. Increased epinephrine and norepinephrine release

Answer: B

Question: Which of the following is the main physiologic response of nitroglycerin administration?

A. Increased preload
B. Increased venous return
C. Decreased total peripheral resistance
D. Decreased left ventricular end systolic volume

Answer: C

Question: What are the changes in 1) pulse pressure and 2) urine flow in a patient with hypovolemic shock?

A. 1) Increase, 2) Increase
B. 1) Decrease, 2) Increase
C. 1) Increase, 2) Decrease
D. 1) Decrease, 2) Decrease

Answer: D

Question: Which of the following **LEAST** likely occurs after an intravenous injection of adrenaline?

A. Increased heart rate
B. Increased blood pressure
C. Increased tone of vagus nerves
D. Increased tone of cardiac sympathetic nerves

Answer: D

Question: Which of the following medications increases myocardial contractility?

A. Low dose of atropine
B. Low dose of dopamine
C. Moderate dose of atropine
D. Moderate dose of dopamine

Answer: D

Question: In an experiment, drug X is given intravenously to a dog. Heart rate increases from 100 to 110 bpm. Subsequently, the vagus nerve is stimulated, but the heart rate is not altered. Which of the following is most likely the drug X?

A. Atropine
B. Dopamine
C. Adrenaline
D. Nitroglycerin

Answer: A

Question: Which of the following is the physiologic response when nitroglycerin is given after atropine has been administered?

A. Increased heart rate
B. Decreased heart rate
C. Increased blood pressure
D. Decreased blood pressure

Answer: D
